# A case report of fulminant cytokine release syndrome complicated by dermatomyositis after the combination therapy with immune checkpoint inhibitors

**DOI:** 10.1097/MD.0000000000019741

**Published:** 2020-04-10

**Authors:** Junichiro Ohira, Michi Kawamoto, Yoshio Sugino, Nobuo Kohara

**Affiliations:** a Department of Neurology, Kobe City Medical Center General Hospital, 650-0047, 2-1-1 Minatojima-Minamimachi, Chuou-ku, Kobe, Hyogo; b Department of Neurology, Kyoto University Hospital, 606-8507, 54 Kawaharacho, Shogoin, Sakyo-ku, Kyoto; c Department of Urology, Kobe City Medical Center General Hospital, 650-0047, 2-1-1 Minatojima-Minamimachi, Chuou-ku, Kobe, Hyogo, Japan.

**Keywords:** cytokine release syndrome, dermatomyositis, immune checkpoint inhibitors, immune-related adverse events

## Abstract

**Introduction:**

Immune-related adverse events (ir-AEs) are increasingly becoming a concern, as immune checkpoint inhibitors (ICIs) are used more frequently. Herein, we present a case of fulminant cytokine release syndrome (CRS) complicated by dermatomyositis after the combination therapy with ICIs.

**Patient concerns:**

A 70-year-old male developed dermatomyositis during the course of treatment with two ICIs, nivolumab and ipilimumab. He was treated by steroid pulse therapy, but the effect was limited. Afterwards, he had acute-onset high fever, hypotension, respiratory failure, impaired consciousness, renal failure, and coagulation abnormality at the same time. C reactive protein (CRP), creatinine kinase (CK), D-dimer, and ferritin levels were considerably elevated: CRP, 24 mg/dL; CK, 40,500 U/L; D-dimer, 290 μg/mL; ferritin, 329,000 ng/mL.

**Diagnosis:**

CRS induced by ICI combination therapy.

**Interventions:**

Given that high fever and elevated CRP level indicated potential sepsis, an antibiotic was used until the confirmation of negative blood cultures. All the simultaneous acute symptoms were supposed to be CRS. He was admitted to the intensive care unit (ICU), and temporary intubation and hemodialysis were needed. Immunosuppressive therapy was reinforced by mycophenolate mofetil together with steroid, and plasma exchange was performed for the elimination of abnormal proteins.

**Outcomes:**

The patient's clinical symptoms and laboratory parameters gradually improved and he was discharged from the ICU in a month.

**Conclusion:**

Fulminant CRS can be induced by ICI combination therapy. As the initial symptoms of CRS resemble sepsis, it is important to consider CRS as a differential diagnosis and to initiate immunosuppressive therapy early when needed. In steroid-resistant cases, early introduction of other immunosuppressive therapy and plasma exchange can be effective.

## 1 Introduction

Immune checkpoint inhibitors (ICIs) have emerged as a novel class of anti-cancer agents. ICIs include the programmed cell death protein 1 (PD-1), its ligand (PD-L1), and the cytotoxic T-lymphocyte associated protein 4 (CTLA-4) inhibitors. The basic mechanism of action of these drugs is to release the brakes of immune regulation and to restore anti-cancer immune response.^1^,^2^ It has been recently demonstrated that combination therapy with nivolumab (a PD-1 inhibitor) and ipilimumab (a CTLA-4 inhibitor) prolonged survival better than standard chemotherapy for the treatment of metastatic renal cell carcinoma.^[3]^ However, various immune-related adverse events (ir-AEs) have been reported to occur during such treatment.^[2]^ The incidence of ir-AEs in melanoma patients was higher during combination therapy than during monotherapy with either nivolumab or ipilimumab^[4]^, and thus ir-AEs are of greater concern in the combination use of ICIs. Neuromuscular ir-AEs sometimes occur and myositis is known to be one of the most frequent ones.^[5]^

Cytokine release syndrome (CRS) is a condition of immune system hyperactivation typically reported in leukemia patients after chimeric antigen receptor engineered T (CAR-T) cell therapy.^6^,^7^,^8^ Although uncommon, severe CRS can occur after the administration of ICIs.^9^,^10^,^11^,^12^,^13^ In this report, we describe a patient with fulminant CRS complicated by dermatomyositis after ICI combination therapy, who temporarily needed intubation and hemodialysis, but recovered after immunosuppressive therapy and plasma exchange followed by intravenous immunoglobulin (IVIg) therapy.

## 2 Case report

An appropriate written informed consent for publication was obtained from the patient and his family.

### 2.1 Patient background before admission

**Figure 1:**
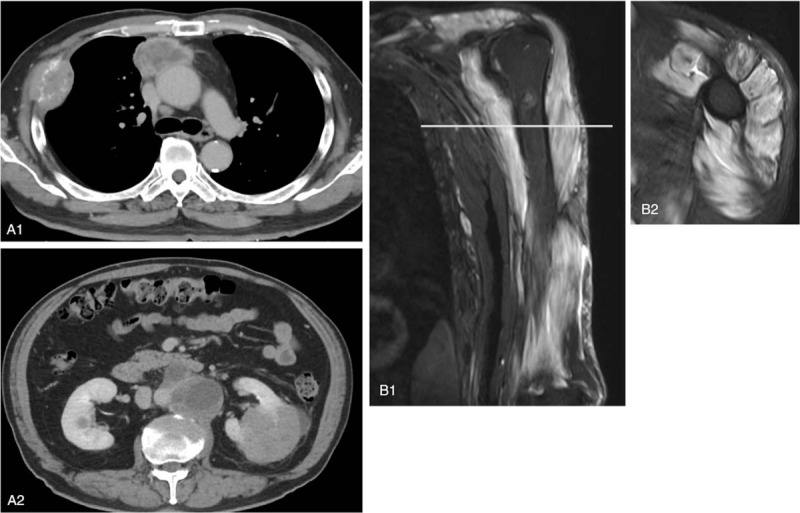
(A) Truncal contrast-enhanced computed tomography at the diagnosis of multiple tumors. (A1) Tumors of mediastinal lymph nodes and the right thoracic wall. (A2) Tumors of the left kidney and para-aortic lymph nodes. (B) Short-tau inversion recovery MRI showing broad hyperintensity in left upper limb muscles. (B1) Coronal section. (B2) Axial section.

**Figure 2:**
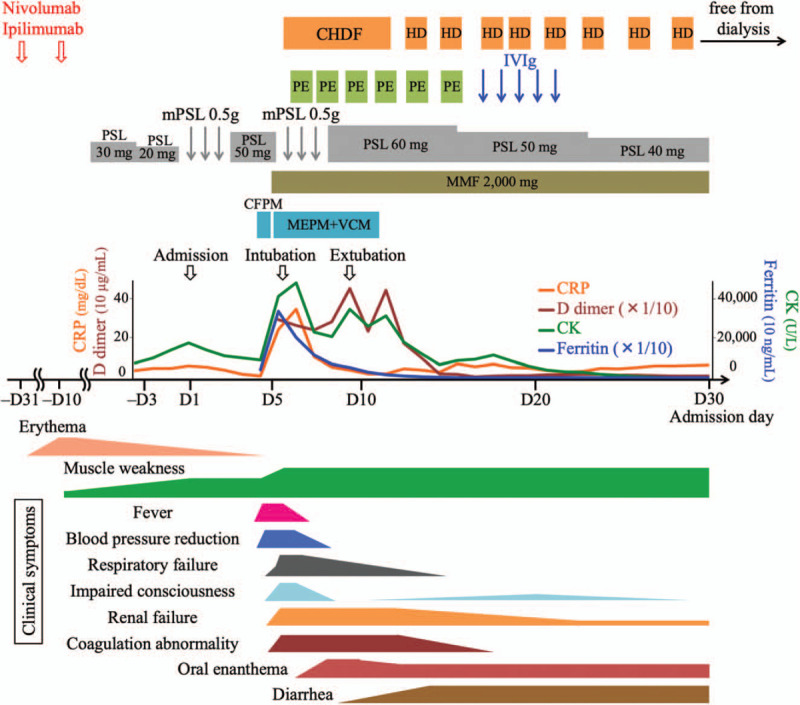
Clinical course after the administration of two immune checkpoint inhibitors (ICIs), nivolumab and ipilimumab. Rash, progressive muscle weakness, and high creatinine kinase level led us to the diagnosis of dermatomyositis induced by ICIs. Steroid therapy failed to prevent acute deterioration of symptoms such as high fever, mild drop in blood pressure, respiratory failure, mildly impaired consciousness, renal failure, and coagulation abnormality. Considerably elevated levels of C reactive protein, creatinine kinase, D-dimer, and ferritin were detected as shown in the graph in the middle. Antibiotic treatment was used until the confirmation that blood cultures were negative. All simultaneous acute symptoms likely represented cytokine release syndrome induced by ICIs. Although temporary intubation and hemodialysis were needed, administration of mycophenolate mofetil together with steroid and plasma exchange followed by intravenous immunoglobulin improved the above symptoms and abnormalities of laboratory parameters. Intractable oral enanthema and diarrhea followed the acute deterioration. Muscle weakness did not improve. CFPM = cefepime, CHDF = continuous hemodiafiltration, CK = creatinine kinase, CRP = C reactive protein, HD = hemodialysis, IVIg = intravenous immunoglobulin, MEPM = meropenem, MMF = mycophenolate mofetil, mPSL = methylprednisolone, PE = plasma exchange, PSL = prednisolone, VCM = vancomycin.

A chest X-ray during a routine medical examination of a 70-year-old male on September 2018 revealed a large tumor in the right lung field. Truncal contrast-enhanced computed tomography (CT) for further evaluation showed multiple tumors in mediastinal lymph nodes, lung, left kidney, para-aortic lymph nodes, and vertebral bodies, as well as in the right thoracic wall, which had been identified in the chest X-ray (Fig. 1A (A1, A2)). The left renal biopsy confirmed the diagnosis of renal cell carcinoma with multiple metastases. It was evaluated as clinical stage 4, T4N1M1 in accordance with TNM classification of Malignant Tumor 8th edition (Union for International Cancer Control, Geneva, Switzerland) and pathological grade 4.^[14]^ As a surgical operation was not applicable, a combination of the ICIs, nivolumab and ipilimumab, was chosen as the initial treatment (Fig. 2).^[3]^ On the following day after the administration of the both drugs, erythema appeared on his dorsal hands and gradually spread to all extremities and body trunk on a daily basis. As the application of steroid ointment was not effective, 30 mg oral prednisolone was administered three weeks after the appearance of erythema. On the same day, nivolumab and ipilimumab were administered for the second time, because erythema was considered to be the only side effect of the first administration. Two days after the second administration, the patient began to have difficulty in elevating his upper limbs and standing up from a squatting position. Although erythema started to improve and oral prednisolone was reduced to 20 mg, he was referred to our Neurology Department by the urologist due to developing muscle weakness 1 week after the second administration. On physical examination, the patient did not have respiratory distress and was alert. He had Gottron sign, shawl sign, and mild erythema on the back, abdomen, and face. Neurological examination revealed proximal limb muscle and truncal weakness without easy fatigability. He had no gait disturbance but had difficulty in sitting up from the supine position. Cranial nerve abnormalities including impairment of ocular movement, ptosis, dysarthria, or dysphasia were not observed. All other neurological findings were normal. The blood test revealed marked elevation of creatinine kinase (CK, 17,386 U/L) (Table 1) and positive for anti-Mi-2 (>150; normal range, 0–52) and anti-transcriptional intermediary factor 1-γ (anti-TIF1-γ) antibodies (50; normal range, 0–31). The patient had a lower titer of anti-Mi-2 antibody (124) and was negative for anti-TIF1-γ antibody before the administration of the ICIs. Anti-aminoacyl tRNA synthetase antibodies and an acetylcholine receptor antibody were negative. Electromyography showed active myopathic changes in the left biceps, right deltoid, and left paraspinal muscles. Low-frequency repetitive stimulation demonstrated no decrement in the left ulnar, facial, and accessory nerves. Magnetic resonance imaging of the left proximal upper limb showed edema and inflammation (Fig. 1B). Therefore, the patient was diagnosed with dermatomyositis, which most likely was an ir-AE (Grade 3, according to the Common Terminology Criteria for Adverse Events [version 5.0])^[15]^ induced by the two ICIs, and admitted to our Neurology Department.

**Table 1:**
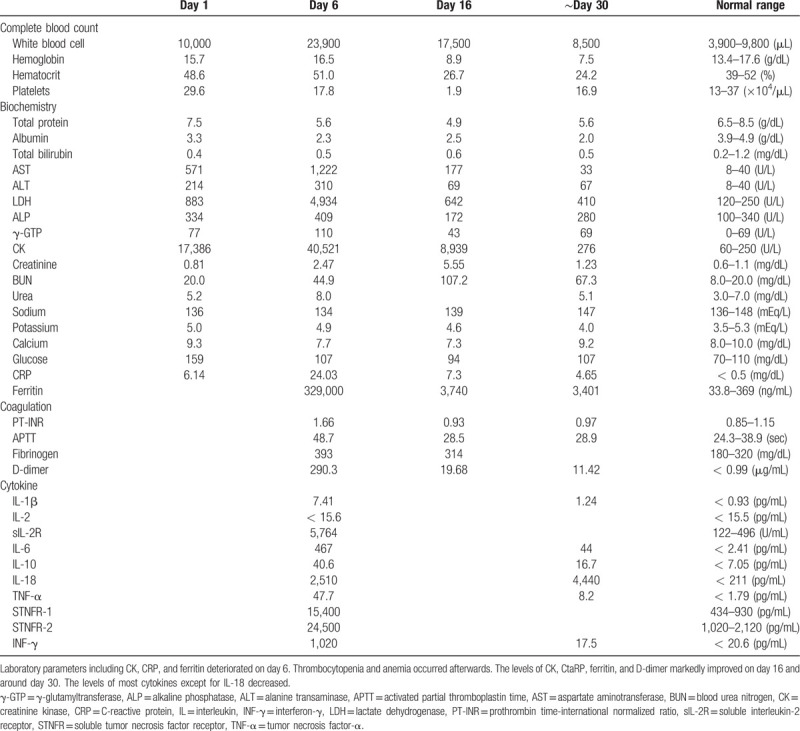
Laboratory parameters on days 1, 6, 16, and around day 30.

### 2.2 Clinical course after admission

As his muscle weakness was progressing even after oral administration of prednisolone at 20 to 30 mg, he was treated with intravenous pulse therapy of 500 mg methylprednisolone for 3 days from the admission day (day 1), followed by 30 mg oral prednisolone (Fig. 2). Both ICIs were discontinued. The reduction in CK level was limited to mild, and muscle weakness remained at the same level. In the evening of day 5, the patient had an acute episode of high fever (39°C) and started shivering. Systolic blood pressure dropped to 100 mmHg from 150 mmHg. As the blood test revealed elevated white blood cell count (27,800/μL) and C reactive protein level (CRP, 8.89 mg/dL), sepsis was suspected, and intravenous administration of cefepime was initiated. Next morning (day 6), the patient had pale extremities, respiratory failure, anuria due to acute renal failure, and mildly impaired consciousness, in addition to high fever. The laboratory tests showed considerable elevation of CRP, CK, D-dimer, and ferritin levels, which indicated disseminated intravascular coagulation and cytokine storm (Table 1). Arterial blood showed lactic acidosis (lactate level, 6.2 mmol/L) and hypoxemia (PaO_2_, 57.4 mmHg). The patient's symptoms were getting worse hourly, and he was moved to the intensive care unit (ICU). His cardiac function was normal and the chest X-ray showed no abnormalities except for the known tumor in the right lung field. He was intubated due to acute respiratory failure and continuous hemodiafiltration (CHDF) was initiated for acute renal failure (Fig. 2). Catecholamine was not needed to keep the blood pressure. All acute events that occurred simultaneously on day 5 were considered to indicate CRS (Grade 4, according to the Common Terminology Criteria for Adverse Events [version 5.0])^[15]^ induced by ICIs, which would be steroid-resistant, rather than sepsis. Therefore, from the ICU admission day immunosuppressive therapy was strengthened with 2000 mg of mycophenolate mofetil (MMF), which is recommended in various steroid refractory ir-AEs, together with second methylprednisolone pulse therapy.^[16]^ In addition, plasma exchange was performed in order to remove abnormal cytokines and replace coagulation factors with fresh frozen plasma. IVIg therapy followed 6 times of plasma exchange. Blood cultures collected before the administration of antibiotics were predictably negative and sepsis was ruled out at this point. The immunosuppressive therapy and plasma exchange were effective. The patient was extubated 3 days after the intubation. CHDF was switched to intermittent hemodialysis, and he got off hemodialysis on day 29 and was discharged from the ICU. Laboratory parameters including CRP, CK, D-dimer, and ferritin levels improved gradually, and the levels of most cytokines decreased (Table 1). Thrombocytopenia gradually developed and platelet count reached the minimum of 19,000/μL on day 16. Bone marrow biopsy revealed mild hemophagocytosis, but megakaryocyte count was normal. Thrombocytopenia slowly improved, possibly as a result of immunotherapy. Anemia gradually progressed and did not improve conceivably due to renal dysfunction. Additional problems that occurred a few days after the acute deterioration were intractable oral enanthema and diarrhea without abdominal pain. Tests for serum β-D glucan, cytomegalovirus antigenemia, and *Clostridium difficile* toxin in the ICU were negative. The biopsy of oral mucosa and tongue ruled out pemphigus vulgaris and pemphigoid, suggesting mucosal damage by the ICIs. Oral enanthema improved around day 60. Colonoscopy revealed mildly white colon mucosa, and colon biopsy showed mild lymphocytic inflammation with edema, which was compatible with colitis induced by ICIs.^17^,^18^ As it was difficult to continue enteral nutrition because of diarrhea, a central venous port was implanted for intravenous hyperalimentation. It was, of course, difficult to administer nivolumab and ipilimumab again due to the fulminant ir-AEs. Multiple tumors slightly enlarged within one month before the administration of the two ICIs, but remained almost the same size for two months after the admission, likely due to the positive effect of the ICIs. The muscle weakness did not improve possibly due to the complication of critical illness. The patient changed hospital for recuperation on day 93.

## 3 Discussion

We present a case of fulminant CRS induced by ICI combination therapy. Dermatomyositis preceded the CRS and was steroid-resistant. Plasma exchange and immunosuppressive therapy with steroid and MMF gradually improved clinical symptoms and laboratory parameters.

Various ir-AEs including neuromuscular disorders have been reported so far in conjunction with ICI use.^2^,^19^ Myositis induced by ICIs (irMyositis) is one of the most common neuromuscular adverse events. Most cases of irMyositis can be successfully treated with steroid therapy^[19]^ and IVIg or plasma exchange can be additional options.^[16]^ In the present case, however, the effect of steroid was limited and fulminant CRS followed irMyositis. Additional plasma exchange and IVIg did not improve muscle weakness. As the rate of fetal myositis is higher in ICI combination therapy than in monotherapy,^[20]^ the effect of steroid was limited in the present case possibly because of the combination therapy.

Diarrhea due to colitis was another ir-AE which occurred in this case. Frequency of diarrhea and colitis are higher in ICI combination therapy than in monotherapy.^[21]^ Gastrointestinal adverse events can occur any time during treatment with ICIs. The management of diarrhea and colitis is not standardized, but in severe cases, ICIs should be permanently discontinued and immunosuppressive therapy with steroid or infliximab is recommended.^[21]^ In the present case, diarrhea did not improve by immunosuppressive therapy including steroid, MMF, and IVIg, inevitably resulting in the implantation of a central venous port. Infectious etiology was ruled out by colonoscopy and examinations for serum β-D glucan, cytomegalovirus antigenemia, and *Clostridium difficile* toxin.

CRS is known to commonly occur after CAR-T cell therapy.^[8]^ The symptoms can vary from mild to severe and include high fever, hypotension, respiratory failure, renal failure, hepatic damage, disseminated intravascular coagulation, and encephalopathy. As the most common initial symptom is high fever, it is often difficult to distinguish CRS from sepsis. Another differential diagnosis is tumor lysis syndrome, which can be diagnosed by laboratory findings. In the present case, several severe symptoms like those mentioned above occurred simultaneously, and blood cultures were negative. These findings strongly supported the diagnosis of CRS.

To the best of our knowledge, there have been only 4 reported cases of CRS after the administration of ICIs (Table 2)^10^,^11^,^12^,^13^. There likely were unreported or missed cases, which might have been misdiagnosed as sepsis. High fever was the first symptoms in all 4 cases, and CRS occurred between the first and fourth administration of ICIs, which was similar timing of the occurrence of other ir-AEs.^[2]^ In the present case, high fever preceded all symptoms about 2 weeks after the second administration of ICIs. The symptoms of the present case were relatively severe compared to other four cases, and the complication of myositis prior to CRS was unique in the present case. Four reported cases had no other immunological disorders before CRS. Severe CRS following irMyositis in the present case might be because of the combination therapy of ICIs, whereas nivolumab monotherapy was used in the other four cases. Treatment of CRS is not established, but tocilizumab, an anti-interleukin 6 receptor antibody, and steroid are currently recommended for CRS after CAR-T cell therapy.^[8]^ In addition, one case report suggested the effectiveness of plasma exchange.^[22]^ As for CRS induced by ICIs, three of four reported cases underwent treatments: high-dose steroid and tocilizumab^[11]^, oral prednisolone^[12]^, and steroid pulse therapy, MMF, and thrombomodulin^[13]^, which improved each clinical symptom at least partially. As preceding dermatomyositis was resistant to steroid therapy in the present case, immunosuppressive therapy was reinforced by MMF in addition to the second steroid pulse therapy, and plasma exchange was performed for the elimination of abnormal coagulation factors, which gradually improved our patient's symptoms.

**Table 2:**
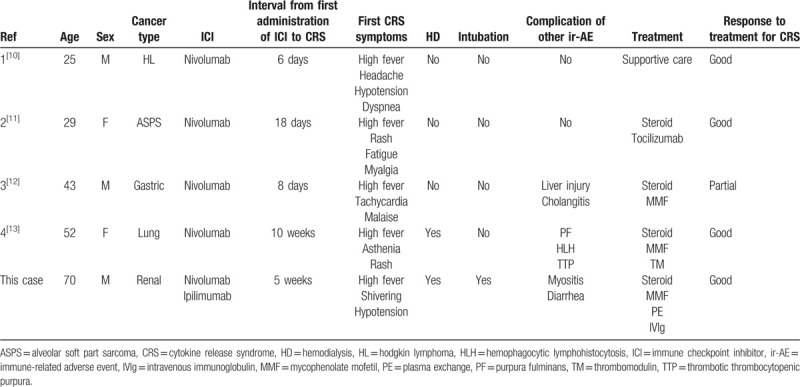
Comparison between four reported cases of CRS after the administration of ICIs and the present case.

The risk factors for CRS after the administration of ICIs are unknown and even those for other relatively common ir-AEs post ICI treatment have not yet been elucidated.^[2]^ It is considered that high disease burden (the percentage of blast cells in bone marrow) in leukemia is one of the most important risk factors for CRS after CAR-T cell therapy.^[8]^ According to this point of view, large size of the tumor that included both primary and metastatic lesions might have contributed to the fulminant CRS in our case.

ICI combination therapy is associated with severe ir-AEs and multiorgan involvement more frequently than monotherapy.^20^,^23^ As mentioned above, risk of fatal myositis and higher grade diarrhea is higher in combination therapy. Notably, fatal ir-AEs tend to occur early after ICI treatment and earlier in combination therapy. (Median time from therapy initiation to symptom onset: 14.5 days in combination therapy and 40 days in monotherapy)^[20]^ In the present case, dermatomyositis (Grade 3) and CRS (Grade 4) occurred 23 days and 35 days after the first administration of ICIs, which were compatible timing with the previous reports.

In conclusion, dermatomyositis and fulminant CRS occurred after the combination therapy with ICIs. As the initial symptoms of CRS resemble sepsis, it is important to consider CRS as a differential diagnosis and to initiate immunosuppressive therapy early when needed. Due to a possible risk of severe CRS, more careful observation of symptoms may be necessary especially in the early phase if ICI combination therapy is performed for the patients with large tumors. In steroid-resistant cases, early introduction of additional immunosuppressive therapy and plasma exchange can be effective.

## Author contributions

**Writing – original draft:** Junichiro Ohira.

**Writing – review & editing:** Michi Kawamoto, Yoshio Sugino, Nobuo Kohara.

## References

[1] Pardoll DM The blockade of immune checkpoints in cancer immunotherapy. Nat Rev Cancer 2012;12:252–64. 22437870 10.1038/nrc3239 PMC4856023

[2] Postow MA Sidlow R Hellmann MD Immune-related adverse events associated with immune checkpoint blockade. N Engl J Med 2018;378:158–68. 29320654 10.1056/NEJMra1703481

[3] Powles T Albiges L Staehler M Updated European Association of Urology guidelines recommendations for the treatment of first-line metastatic clear cell renal cancer. Eur Urol 2017;73:311–5. 29223605 10.1016/j.eururo.2017.11.016

[4] Larkin J Chiarion-Sileni V Gonzalez R Combined nivolumab and ipilimumab or monotherapy in untreated melanoma. N Engl J Med 2015;373:23–34. 26027431 10.1056/NEJMoa1504030 PMC5698905

[5] Moreira A Loquai C Pföhler C Myositis and neuromuscular side-effects induced by immune checkpoint inhibitors. Eur J Cancer 2019;106:12–23. 30453170 10.1016/j.ejca.2018.09.033

[6] Lee DW Gardner R Porter DL Current concepts in the diagnosis and management of cytokine release syndrome. Blood 2014;124:188–95. 24876563 10.1182/blood-2014-05-552729 PMC4093680

[7] Grupp SA Kalos M Barrett D Chimeric antigen receptor-modified T cells for acute lymphoid leukemia. N Engl J Med 2013;368:1509–18. 23527958 10.1056/NEJMoa1215134 PMC4058440

[8] Shimabukuro-Vornhagen A Gödel P Subklewe M Cytokine release syndrome. J Immunother Cancer 2018;6:56. 29907163 10.1186/s40425-018-0343-9 PMC6003181

[9] Kähler KC Hassel JC Heinzerling L Management of side effects of immune checkpoint blockade by anti-CTLA-4 and anti-PD-1 antibodies in metastatic melanoma. J Dtsch Dermatol Ges 2016;14:662–81. 10.1111/ddg.13047 27373241

[10] Zhao L Yang Y Li W Nivolumab-induced cytokine-release syndrome in relapsed/refractory Hodgkin's lymphoma: a case report and literature review. Immunotherapy 2018;10:913–7. 30149764 10.2217/imt-2018-0025

[11] Rotz SJ Leino D Szabo S Severe cytokine release syndrome in a patient receiving PD-1-directed therapy. Pediatr Blood Cancer 2017;64:e26642. 10.1002/pbc.26642 28544595

[12] Oda H Ishihara M Miyahara Y First case of cytokine release syndrome after nivolumab for gastric cancer. Case Rep Oncol 2019;12:147–56. 31043953 10.1159/000496933 PMC6477485

[13] Honjo O Kubo T Sugaya F Severe cytokine release syndrome resulting in purpura fulminans despite successful response to nivolumab therapy in a patient with pleomorphic carcinoma of the lung: a case report. J Immunother Cancer 2019;7:97. 30944043 10.1186/s40425-019-0582-4 PMC6448268

[14] Moch H The WHO/ISUP grading system for renal carcinoma. Pathologe 2016;37:355–60. 27271258 10.1007/s00292-016-0171-y

[15] National Cancer Institute: the Common Terminology Criteria for Adverse Events (CTCAE) 5.0.

[16] Brahmer JR Lacchetti C Schneider BJ Management of immune-related adverse events in patients treated with immune checkpoint inhibitor therapy: American Society of Clinical Oncology Clinical Practice Guideline. J Clin Oncol 2018;36:1714–68. 29442540 10.1200/JCO.2017.77.6385 PMC6481621

[17] Michot JM Bigenwald C Champiat S Immune-related adverse events with immune checkpoint blockade: a comprehensive review. Eur J Cancer 2016;54:139–48. 26765102 10.1016/j.ejca.2015.11.016

[18] Beck KE Blansfield JA Tran KQ Enterocolitis in patients with cancer after antibody blockade of cytotoxic T-lymphocyte-associated antigen 4. J Clin Oncol 2006;24:2283–9. 16710025 10.1200/JCO.2005.04.5716 PMC2140223

[19] Fellner A Makranz C Lotem M Neurologic complications of immune checkpoint inhibitors. J Neurooncol 2018;137:601–9. 29332184 10.1007/s11060-018-2752-5

[20] Wang DY Salem J-E Cohen JV Fatal toxic effects associated with immune checkpoint inhibitors: a systematic review and meta-analysis. JAMA Oncol 2018;4:1721–8. 30242316 10.1001/jamaoncol.2018.3923 PMC6440712

[21] Tandon P Bourassa-Blanchette S Bishay K The risk of diarrhea and colitis in patients with advanced melanoma undergoing immune checkpoint inhibitor therapy: a systematic review and meta-analysis. J Immunother 2018;41:101–8. 29401166 10.1097/CJI.0000000000000213

[22] Xiao X He X Li Q Plasma exchange can be an alternative therapeutic modality for severe cytokine release syndrome after chimeric antigen receptor-T cell infusion: a case report. Clin Cancer Res 2019;25:29–34. 30322878 10.1158/1078-0432.CCR-18-1379

[23] Zhou S Khanal S Zhang H Risk of immune-related adverse events associated with ipilimumab-plus-nivolumab and nivolumab therapy in cancer patients. Ther Clin Risk Manag 2019;15:211–21. 30774357 10.2147/TCRM.S193338 PMC6362938

